# Short-Term Impact of Video-Assisted Thoracoscopic Surgery on Lung Function, Physical Function, and Quality of Life

**DOI:** 10.3390/healthcare9020136

**Published:** 2021-02-01

**Authors:** Yoshiteru Akezaki, Eiji Nakata, Ritsuko Tominaga, Orie Iwata, Juichi Kawakami, Tetsuya Tsuji, Tsuyoshi Ueno, Motohiro Yamashita, Shinsuke Sugihara

**Affiliations:** 1 Division of Physical Therapy, Kochi Professional University of Rehabilitation, Kochi 781-1102, Japan; akezakiteru@yahoo.co.jp; 2 Department of Orthopaedic Surgery, Okayama University Hospital, Okayama 700-8558, Japan; 3 Department of Rehabilitation Medicine, National Hospital Organization Shikoku Cancer Center, Ehime 791-0280, Japan; tominaga.ritsuko.fk@mail.hosp.go.jp (R.T.); sugihara.shinsuke.rk@mail.hosp.go.jp (S.S.); 4 Department of Rehabilitation Medicine, National Hospital Organization Tokushima Hospital, Tokushima 776-8585, Japan; iwata.orie.jv@mail.hosp.go.jp; 5 Department of Rehabilitation Medicine, Shiga Prefectural Rehabilitation Center, Shiga 524-0022, Japan; juichi-k@venus.dti.ne.jp; 6 Department of Rehabilitation Medicine, Keio University School of Medicine, Tokyo 160-8582, Japan; cxa01423@nifty.com; 7 Department of Thoracic Surgery, National Hospital Organization Shikoku Cancer Center, Ehime 791-0280, Japan; ueno.tsuyoshi.qz@mail.hosp.go.jp (T.U.); yamashita.motohiro.tr@mail.hosp.go.jp (M.Y.)

**Keywords:** lung cancer, surgery, physical function, lung function, quality of life

## Abstract

*Background*: Video-assisted thoracoscopic surgery (VATS) has been increasingly used as an approach for lung lobectomy. However, the recovery of respiratory and physical function may be insufficient at discharge because the average length of hospital stay is decreasing after surgery. In this study, we investigated the changes in physical function, lung function, and quality of life (QOL) of lung cancer patients after VATS, and factors for QOL were also evaluated. Methods: The subjects of this study were 41 consecutive patients who underwent video-assisted lung lobectomy for lung cancer. Rehabilitation was performed both before and after surgery. Lung function testing, physical function testing (timed up and go test (TUG) and the 30-s chair-stand test (CS-30)), and QOL (EORTC QLQ-C30) were measured before and 1 week after surgery. Results: Postoperative VC recovered to 76.3% ± 15.6% 1 week after surgery. TUG, CS-30, and QOL were significantly worse after surgery (*p* < 0.05). Lung function and physical function were found to affect QOL. Postoperative complications included pneumonia in 1 patient. There were no patients who discontinued rehabilitation. Conclusion: Our rehabilitation program was safe and useful for patients after VATS.

## 1. Introduction

Lung cancer is the most prevalent cancer diagnosed worldwide and is associated with the highest mortality [1,2]. Modern surgical treatment includes both minimally invasive surgery, e.g., video-assisted thoracoscopic surgery (VATS) and open surgery, such as thoracotomy. VATS has been increasingly used as an approach for lung lobectomy. The reported benefits of this technique when compared with open procedures include less postoperative pain [3,4], reduced length of hospital stay and loss of lung function [5], and better postoperative quality of life (QOL) [3]. Regarding rehabilitation for VATS patients, enhanced recovery program including rehabilitation has been shown to have no effect on cardiopulmonary complications, 30- and 90-day mortality, length of stay, and readmissions [6]. On the other hand, other studies have shown that most of VATS lobectomy patients demonstrated conditions potentially amenable to rehabilitation [7]. Therefore, it has been reported that all patients need to receive routine postoperative rehabilitation evaluations to identify problems early and correctly [7].

After VATS and thoracotomy, it takes 3–6 months for patients to recover more than 80% of vital capacity (VC) [8]. Regarding physical function, postoperative patients are prone to a decline in physical function due to bed rest and immobility following surgery. The recovery of respiratory and physical function may be insufficient at discharge compared to the preoperative level because the length of stay is shorter in lung cancer patients following VATS than after thoracotomy. This has an effect on the postoperative quality of life (QOL) of these patients. However, there are no studies of the changes of physical abilities and QOL from before to a short time after VATS. Clarifying the recovery of physical function and QOL at discharge is considered useful for medical staff to give activities of daily living (ADL) guidance and plan exercise for patients.

In this study, we investigated the changes in physical function, lung function, and QOL of lung cancer patients after VATS, and factors for QOL were also evaluated.

## 2. Methods

### 2.1. Study Design

This was a retrospective, observational study of lung and physical function and QOL in lung cancer survivors before and one week after VATS.

### 2.2. Patients and Methods

**Figure 1:**
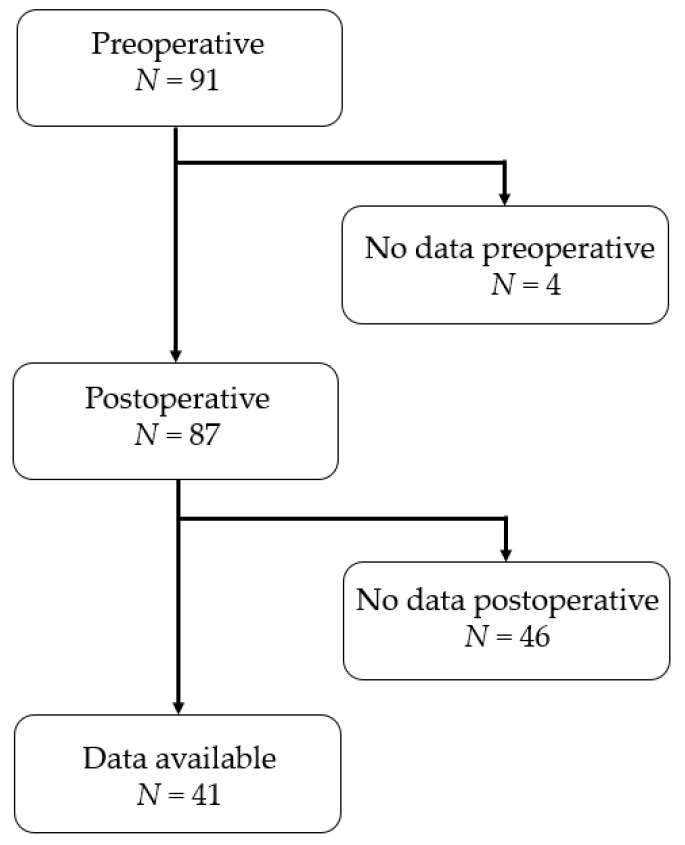
Flowchart of the study.

Among 91 patients who had undergone video-assisted lung lobectomy for lung cancer, 41 whose measurements were available from before and after surgery were included (Figure 1). The exclusion criteria were decreased cognitive function.

Lung function testing, physical function (timed up and go test (TUG) [9] and 30-s chair-stand test (CS-30) [10]), QOL (EORTC QLQ-C30) [11], postoperative pulmonary complications, and the day of starting walking after surgery were investigated. Measurements were performed before and 1 week after surgery.

### 2.3. Video-Assisted Thoracoscopic Surgery

Four surgeons certified with Japanese association of respiratory surgery performed these VATS operations. Each surgeon had experience of more than 60 cases per year for more than 3 years. Surgeons performed VATS via a small 3–5 cm incision in the fourth or fifth intercostal space and 2 or 3 ports. Surgeons operated under complete monitor vision.

### 2.4. Rehabilitation Program

The preoperative program consisted of muscle-strengthening exercises including squatting and calf raise exercises, stretching including the upper limb, lower limb, and trunk, abdominal respiration, guidance in airway clearance including cough techniques, and respiratory muscle training with incentive spirometry (Coach2^®^, Smiths Medical Inc., Minneapolis, MN, USA), walking (more than 20 min per day), and aerobic exercise (15 to 30 min per day). 

On postoperative day (POD) 1, guidance in airway clearance, abdominal respiration, sitting, standing, and standing still were started. On POD 2, walking was started. On POD 3, aerobic exercise was started. On POD 4, stair climbing and descending were performed, and physical activities were increased depending on the patient’s physical condition. The postoperative rehabilitation was 20–40 min per day. The timing of mobilization changed according to the physical status of the patient for a few days. 

The preoperative and postoperative exercise intensity was set to the Borg Category-Ratio scale (Borg CR-10) of 3–5 points. The preoperative and postoperative rehabilitation was given under the guidance of a physiotherapist. 

Regarding the criteria at discharge, the doctor judged if the patients were following an uneventful postoperative course from chest X-ray, blood data, and the physical condition and careful examination of the risk of developing postoperative complications.

### 2.5. Lung Function

Forced vital capacity (FVC), %FVC (FVC/predicted), forced expiratory volume in 1 s (FEV1), FEV1% (FEV1/FVC), %FEV1 (FEV1 % predicted) were evaluated with the patients seated using a spirometer (Spiro Shift sp-470, Fukuda Denshi, Tokyo, Japan). Predicted FVC was calculated using the equation developed by the Japanese Respiratory Society [12].

The predicted postoperative VC was evaluated as follows: predicted postoperative VC = preoperative VC × (residual segment number/total segment number) [13]. The recovery rate of postoperative VC is the ratio of postoperative VC to predicted postoperative VC.

### 2.6. Physical Function

The TUG [9] was evaluated twice at an interval of 1 min, with the fastest values adopted as the representative values. The time required to stand up from a chair when sitting, circle around a landmark set 3 m ahead, and sit back down on the chair as fast as possible was evaluated. The starting point for measurement was the time when the back of the patient left the backrest of the chair, with the end measurement point being when the patient sat back down in the chair. 

Lower extremity muscle endurance was evaluated using the CS-30 [10]. The chair-stand test was administered using a seat height of 40 cm. The patients were encouraged to stand up from a chair as many times as possible without stopping, keeping the arms folded across the chest. At the signal “go”, the participant rose to a full stand (body erect and straight) and then returned back to the initial seated position. Only full standing positions were counted. The analysis used the number of repetitions performed with the arms folded across the chest.

### 2.7. Health-Related Quality of Life

Cancer-specific health-related QOL was assessed by the EORTC QOL Questionnaire C30 [11]. The questionnaire incorporated five functional scales (physical, role, cognitive, emotional, and social functions), three symptom scales (fatigue, pain, nausea, and vomiting), global health status, and a number of single items assessing additional symptoms (dyspnea, loss of appetite, insomnia, constipation, and diarrhea) and the perceived financial impact of the disease. All scores resulted in a value between 0 and 100; high scores on functional and global QOL imply a high level of function, while a higher score on the symptom scales indicates greater problems [14,15,16].

### 2.8. Statistical Analysis

For lung function, physical function, and QOL, pre- and postoperative comparisons were analysed using paired *t*-tests and Wilcoxon signed-rank tests. To assess the factors affecting QOL after surgery, stepwise multiple regression analyses were constructed using the items of QLQ-C30 as independent variables and the data of age, presence or absence of chronic obstructive pulmonary disease (COPD), TUG after surgery, CS-30 after surgery, recovery rate of postoperative VC, day of start walking after surgery, FVC, %FVC, FEV1, and FEV1% after surgery as the dependent variables.

SPSS software version 22.0 (IBM, Tokyo, Japan) was used to analyze the collected data, and differences were considered significant at a *p*-value < 0.05.

## 3. Results

### 3.1. Socio-Demographic and Clinical Characteristics

A total of 41 consecutive patients (25 men, 16 women) were recruited. The average ± standard deviation (SD) age at the time of the study was 66.3 ± 9.7 years. The average ± SD body mass index (BMI) was 23.2 ± 3.0 kg/m^2^. Table 1 shows comorbidities. Some patients had multiple comorbidities.

Operative procedures were right upper lobectomy in 12 patients, right middle lobectomy in 3 patients, right lower lobectomy in 9 patients, left upper lobectomy in 9 patients, left lower lobectomy in 6 patients, and left upper and lower lobectomies in 2 patients.

The median period of preoperative rehabilitation was 3 (1–13) days. The median period of postoperative chest tube placement was 2 (1–5) days. The patients’ median day of starting walking after surgery was 2 (1–2) days. The patients’ median length of stay after surgery was 8 (6–25) days. All patients achieved independent gait at 1 week after surgery. Postoperative rehabilitation was possible for 37 patients as planned. Four patients could not carry out as prescribed. Their start of aerobic exercise and stair climbing were delayed according to their conditions and took place on the POD 4, 5, 6, and 22, respectively. Postoperative complications included pneumonia in 1 patient.

### 3.2. Differences in Lung Function before and after VATS

The average recovery rate of postoperative VC was 76.3% ± 15.6%. Table 2 shows lung and physical function. Postoperatively, FVC, FVC/predicted, FEV1, FEV1/FVC, and FEV1% predicted were also decreased significantly.

### 3.3. Differences in Physical Function before and after VATS

The TUG was significantly longer (*p* < 0.05), and the number of repetitions in the CS-30 was significantly less after surgery (*p* < 0.05).

### 3.4. Differences in Health-Related QOL before and after VATS

The pre- and postoperative QLQ-C30 results are shown in Table 3. After surgery, global health status/QOL scale, physical, role, social, and fatigue in the functional scales and pain, dyspnoea, sleep disturbance, appetite loss, and constipation in the symptoms scale were significantly worse compared with the preoperative evaluation (*p* < 0.05).

### 3.5. Factors Affecting Postoperative QOL

Table 4 shows the results of the multiple regression analyses. Stepwise multiple regression analyses showed that the presence of COPD was associated with global health status (*p* < 0.05, R^2^ = 0.085). The presence of COPD and the CS-30 results were associated with QOL physical (*p* < 0.05, R^2^ = 0.277). The FVC/predicted after surgery was associated with QOL role (*p* < 0.05, R^2^ = 0.195). The recovery rate of postoperative VC was associated with QOL cognitive (*p* < 0.05, R^2^ = 0.148). The FVC/predicted after surgery was associated with QOL dyspnea (*p* < 0.05, R^2^ = 0.100). The results of the CS-30 were associated with appetite loss (*p* < 0.05, R^2^ = 0.174).

## 4. Discussion

Our study investigated the changes in physical function, lung function, and QOL of lung cancer patients after VATS, and factors for QOL were also evaluated. The results showed that VATS had a negative short-term impact on physical function and QOL, and lung function and physical function was found to affect QOL in patients after VATS. 

The percentage decreases in the VC after VATS and thoracotomy have been reported to be 68.8% [8] and 69.1% [17] at 1 week after surgery and 70.5% [8] and 69.0% [17] at two weeks after surgery. VC recovery rate of 76.3% in the present study was higher than previously reported studies, although a direct comparison is not possible because patients of this study were only VATS. VC at the time of 6 months after VATS was 88% of the preoperative value [8]. Therefore, we have thought that the patients of this study will gradually improve by 6 months after discharge.

Atelectasis following lung resection has been described as a common postoperative complication, with an incidence of 1–20% [18,19,20,21]. In the present study, the incidence of postoperative complications was only 2.0% (1 of 41 patients). Preoperative rehabilitation and postoperative early mobilization were emphasized in the present study because mobilization forces the patient to perform more frequent and deeper sighing respiration and promote airway clearance [22] than with respiratory exercises in bed [14]. Therefore, good results could be obtained. Furthermore, a need for long-term rehabilitation during hospitalization is suggested for patients with postoperative complications, even in VATS patients.

In the present study, the TUG and CS-30 were used to evaluate physical function and walking ability. The TUG is a clinical assessment of balance and mobility [23]. It is also a reliable tool to evaluate agility, because it involves walking and changing direction [24]. The CS-30 was used to measure lower body strength [10]. This test has been validated for measurement of lower body strength in older adults with COPD [25]. In the present study, the TUG was significantly longer, and the number of repetitions of the CS-30 was significantly lower after surgery. These results mean that patients had decreased lower limb muscular strength and balance ability postoperatively. Although patients carried out a rehabilitation program for two to 3 weeks before surgery and performed intensive early mobilization from the early postoperative period, the decline of physical performance could not be prevented. Although the invasion was minimal after VATS, we have thought that the physical function and walking ability of the patients decreased after VATS because their physical activity decreased due to pain and thoracic drain placement. As with postoperative patients with other diseases, the importance of an early increase in physical activity has been shown from the study with VATS patients [7]. This study has confirmed the previous study, and we, therefore, consider the importance of early rehabilitation.

It was previously reported that physical health-related QOL was significantly lower even six months after lung surgery compared to preoperatively [26]. However, there have been no previous studies of the short-term impact of VATS. In the present study, global health status, physical, role, and social, except the emotional and cognitive items of QLQ-C30, decreased significantly 1 week after surgery. Concerning the symptom scale, fatigue, pain, dyspnoea, insomnia, appetite loss, and constipation showed significant decreases postoperatively compared with preoperative levels. Although the reported benefits of VATS include reduced postoperative pain [27], the present findings confirmed that negative symptoms were not sufficiently improved 1 week after surgery.

As the result of examining factors affecting postoperative QOL, global health status, physical, role, cognitive, dyspnea, and appetite loss were identified on stepwise multiple regression analyses. Declining lung function, including the presence or absence of COPD, %VC after surgery, and the recovery rate of postoperative VC may have a negative effect on global health status, physical, role, and cognitive among the items of the QLQ-C30 and dyspnea. It was suggested that postoperative QOL and lung function affected each other. Physical and appetite loss were associated with the CS-30. Patients with higher lower extremity muscle endurance might have better physical and appetite recovery.

### Study Limitations

There are some limitations associated with the present study. First, the TUG and CS-30 were used to evaluate physical function, but exercise tolerance tests such as the 6-min walk distance or shuttle walking were not performed. Second, it is necessary to examine not only the short-term, but also the long-term impact of VATS longitudinally. Third, patients are limited to patients of VATS and our study cannot be compared with patients of thoracotomy. Fourth, the effect of pain on postoperative lung function and physical function has not been investigated in this study. Fifth, there was no control group without rehabilitation in the present study, which limits the conclusions about the effects of the intervention. Further research is needed to examine these issues.

## 5. Conclusions

Lung function, physical function, and QOL had not recovered sufficiently at discharge, even though preoperative and early postoperative rehabilitation was performed. However, VC recovery rate and the incidence of postoperative complications was better than previously reported studies, and no patients discontinued rehabilitation. Therefore, our rehabilitation program was safe and useful for patients after VATS.

## Figures and Tables

**Table 1:** Comorbidities of Patient.

Comorbidities	N
Chronic obstructive pulmonary disease	
GOLD stage I	9
GOLD stage II	7
Hypertension	16
Mitral insufficiency	10
Aortic insufficiency	7
Diabetes	7
Angina pectoris	6
Ischemic heart disease	5
Dyslipidemia	6
Tricuspid regurgitation	2
Interstitial pneumonia	1
Congestive heart failure	1

GOLD, Global Initiative for Chronic Obstructive Lung Disease.

**Table 2:** Comparisons of lung function and physical function before and after surgery for lung cancer.

Item	Preoperative		Postoperative		*p* Value
	Mean (SD)	Median	Mean (SD)	Median	
FVC in L (L)	2.5 (0.6)	2.4	1.7 (0.5)	1.7	*p* < 0.001
FVC/pred (%)	88.2 (15.1)	87.0	62.2 (14.8)	61.0	*p* < 0.001
FEV1 in L (L)	1.9 (0.5)	1.9	1.3 (0.3)	1.2	*p* < 0.001
FEV1/FVC (%)	77.6 (8.7)	79.6	76.6 (8.9)	78.9	0.367
FEV1% pred (%)	84.7 (18.1)	84.0	58.3 (15.6)	55.0	*p* < 0.001
TUG (s)	6.2 (1.2)	6.0	6.7 (1.3)	7.0	0.006
CS-30 (number of times)	16.1 (4.4)	16	15.1 (4.3)	14	0.020

FVC, forced vital capacity; pred, predicted; FEV1, forced expiratory volume in 1 s; FEV1%, ratio of FEV1 to FVC; TUG, timed up and go test; CS-30, 30-s chair-stand test.

**Table 3:** Comparison of QLQ-C30 between pre- and postoperative lung cancer.

Item	Preoperative		Postoperative		*p*-Value
	Mean (SD)	Median	Mean (SD)	Median	
Global health status/QOL	68.9 (20.9)	667	58.9 (20.8)	50.0	0.003
Physical function	91.9 (9.9)	93.3	82.0 (13.1)	86.7	*p* < 0.001
Role function	91.5 (16.3)	100	72.9 (24.7)	66.7	0.001
Emotional function	76.0 (20.3)	75.0	78.0 (20.5)	75.0	0.455
Cognitive function	84.6 (16.4)	83.3	81.3 (18.0)	83.3	0.344
Social function	91.5 (15.0)	100	81.3 (22.7)	83.3	0.005
Fatigue	17.1 (16.7)	11.1	32.2 (19.8)	33.3	0.001
Nausea/vomiting	0.8 (3.6)	0	3.7 (10.2)	0	0.084
Pain	7.3 (14.0)	0	28.5 (16.8)	33.3	*p* < 0.001
Dyspnea	9.8 (17.1)	0	31.7 (21.0)	33.3	*p* < 0.001
Insomnia/sleep	19.5 (31.6)	0	30.9 (25.2)	33.3	0.008
Appetite loss	10.6 (21.7)	0	22.0 (26.5)	0	0.022
Constipation	13.8 (22.3)	0	22.8 (30.2)	0	0.033
Diarrhea	5.7 (12.7)	0	7.3 (14.0)	0	0.527
Financial hardship related to illness	8.9 (15.0)	0	13.8 (21.0)	0	0.134

QOL, quality of life.

**Table 4:** Results of stepwise multiple regression analyses.

Item	Included Variable ^†^	*β*	Adjusted R^2^	*p* Value
Global health status/QOL	Presence or absence of COPD	−13.813	0.085	0.036
Physical	Presence or absence of COPD CS30	−13.247 1.208	0.277	0.001 0.007
Role	FVC/predicted after surgery	0.769	0.195	0.003
Cognitive	Recovery rate of postoperative VC	−39.300	0.148	0.008
Dyspnea	FVC/predicted after surgery	−0.496	0.100	0.025
Appetite loss	CS30	−2.703	0.174	0.004
Constipation	Recovery rate of postoperative VC Day of start walking after surgery	88.390 35.801	0.269	*p* < 0.001 0.010
Diarrhea	Age	0.650	0.182	0.003

^†^, Variables were selected by backward stepwise multiple regression models; QOL, quality of life; COPD, chronic obstructive pulmonary disease; CS-30, 30-s chair-stand test; FVC, forced vital capacity; VC, vital capacity; *β*, standardized regression coefficient.

## Data Availability

The data presented in this study are available on request from the corresponding author. The data are not publicly available due to Participant’s personal information.
